# Copper Tridentate Schiff Base Complex Supported on SBA-15 as Efficient Nanocatalyst for Three-Component Reactions under Solventless Conditions

**DOI:** 10.3390/ma11122458

**Published:** 2018-12-04

**Authors:** Elham Sadat Diarjani, Fatemeh Rajabi, Asieh Yahyazadeh, Alain R. Puente-Santiago, Rafael Luque

**Affiliations:** 1 Department of Science, Payame Noor University, P.O. Box 19395-4697, Tehran 19569, Iran; diarjanielham@gmail.com; 2 Department of Chemistry, University of Guilan, P.O. Box 41335-1914, Rasht, Iran; yahyazadeh@guilan.ac.ir; 3 Department of Organic Chemistry, University of Cordoba, Campus de Rabanales, Edificio Marie Curie (C-3), Ctra Nnal IV-A, Km 396, E14014 Cordoba, Spain; apuentesantiago@gmail.com; 4 Peoples Friendship University of Russia (RUDN University), 6 Miklukho-Maklaya str., 117198 Moscow, Russia

**Keywords:** copper Schiff base complex, SBA-15 dihydropyrimidinones, solvent-free reaction, Biginelli reaction

## Abstract

The anchorage of a supported copper Schiff base complex on SBA-15 materials provides highly efficient heterogeneous catalysts towards the solvent-free synthesis of dihydropyrimidinones derivatives via the Biginelli condensation reaction. The novel nanocatalysts exhibited a highly ordered mesostructure with a surface area of 346 m^2^g^−1^ and an average pore diameter of 8.6 nm. Additionally, the supported copper nanocatalysts were reused at least ten times, remaining almost unchanged from the initial activity. Both the mesoporous scaffold and the tridentate Schiff base ligand contributed to the stabilization of copper species.

## 1. Introduction

**Figure 1:**
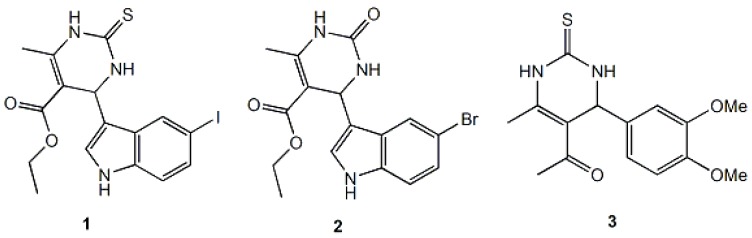
Examples of pharmacologically active dihydropyrimidinones (DHPMs).

Dihydropyrimidinones (DHPMs) have received considerable attention due to their desirable biological properties, making them useful candidates for a myriad of biomedical and clinical applications [1,2,3,4,5,6,7,8]. Among them, a number of dihydropyrimidines molecules featuring pharmacological activities can be observed in Figure 1. In this regard, the development of suitable synthetic protocols for the synthesis of the mentioned molecules constitutes a significant research topic nowadays.

The conventional synthesis of DHPMs was reported as a Biginelli condensation with a multi-component reaction of *β*-dicarbonyl compounds, aromatic aldehydes, and urea using very acidic environments [9]. Due to the distinct biological and pharmacological activity of DHPMs, synthetic methodologies for the Biginelli condensation have recently been developed, including solvent free synthesis [10], microwave irradiation [11], ultrasound radiation [12], visible light irradiation [13], Bronsted and Lewis acids [14], ionic liquids [15], enzymatic catalysts [16], and solid acid catalysts [17]. However, despite considerable progress in this area, a number of these protocols still have several limitations, including the high cost of the materials, significant amounts of generated side-products, the use of toxic solvents, and undesired reaction conditions. Moreover, homogeneous developed catalysts have inherent issues associated with separation or product recovery, product contamination by residual catalyst, or metal species which hinder potential scale-up processes to industry applications. In catalysis, the life-time (recyclability) of a given catalyst is another crucial parameter. Therefore, to develop improved reusable catalytic systems in terms of industrial application, practical simplicity, economic viability, and sustainability is of utmost importance for the synthesis of DHPMs. A suitable strategy towards more sustainable processes relates to the immobilization of homogenous catalysts onto solid supports including silica, zeolites, clays, organic polymers, and organic-inorganic mesoporous materials. Mesoporous silica materials, especially SBA-15, are highly desirable for catalytic applications due to the possibility of high surface areas and porosity, and narrow and uniform pore size distributions which increase both reactivity and selectivity in catalytic reactions, together with a high stability, the possibility to anchor functional groups with a well-established surface chemistry, no swelling biocompatibility, and low toxicity.

In this work, a nanocatalyst based on supported copper Schiff base complex on SBA-15 (Cu@SBA-15) was employed for a three-component coupling Biginelli reaction to their corresponding dihydropyrimidinones. The copper Schiff base complex immobilized on the mesoporous materials displayed good performance and stability under the investigated reaction conditions. The proposed synthetic methodology is simple, low-cost, and eco-environmentally friendly and could be easily translated to industrial applications.

## 2. Materials and Methods

### 2.1. Preparation of L1@SBA-15

SBA-15 was synthesized based on previous reports [18] and activated with 6 M HCl under reflux conditions for 12 h. The suspension was filtered, washed with deionized water until the filtrate became neutral, and dried under vaccum oven (Labplant UK Ltd., North Yorkshire, UK) at 60 °C for 10 h. Amino functionalized SBA-15 was synthesized by refluxing of activated SBA-15 (2.0 g), dropwise adding of N-(2-Aminoethyl)-3-(trimethoxysilyl) propylamine in 50 mL dry toluene. The mixture was refluxed in toluene with continuous removal of water using a Dean-Stark trap for 24 h. The slurry was filtered off, and the resulting solid, L1@SBA-15, was washed with excess amounts of hot toluene and ethanol to remove unreacted diamino silane precursor. It was dried in the vacuum oven at 60 °C for 10 h to furnish L1@SBA-15 at a loading ca. 0.55 mmol·g^−1^ (as determined by TGA analysis).

### 2.2. Preparation of L2@SBA-15

One mmol (0.107 gr) of pyridine-2 carbaldehyde was added to the stirring suspension of 1.0 g L1@SBA-15 in methanol (50 mL). The reaction mixture was refluxed for 24 h. Subsequently, the resulting yellow-colored solid was filtered, washed with excess methanol, and dried under vacuum at 60 °C for 10 h to establish L2@SBA-15at a loading ca. 0.48 mmol·g^−1^ (as determined by TGA analysis).

### 2.3. Preparation of Cu@SBA-15

We added 0.145 g (0.8 mmol) of Cu(II) acetate hydrate to the stirring suspension of L1@SBA-15 (4.6 g) in methanol (50 mL), and refluxed for 24 h. The reaction mixture colour changed from yellow to green. The resultant green-colored solid was filtered, washed with a large volume of methanol, and dried in an oven overnight at 60 °C to furnish the corresponding nanocatalyst Cu@SBA-15 at a loading ca. 0.41 ± 0.01 mmol·g^−1^ (as determined by TGA analysis and atomic absorption spectroscopy (AAS)).

### 2.4. Preparation of 3,4-Dihydropyrimidin-2(1H)-One

In a 50-mL flask, aldehyde (5 mmol), *β*-dicarbonyl compound (5 mmol), urea (6 mmol), and Cu@SBA-15 (10 mg, 0.02 mmol) was added and stirred at 100 °C for 5–10 min under solvent-free conditions. Then hot ethanol was added to the mixture, and the Cu@SBA-15 nanocatalyst was separated by filtration. To test the reusability of the catalyst, after first reaction run, Cu@SBA-15 nanocatalyst was filtered from the reaction mixture. Then, the catalyst was washed with water and ethanol, dried in vacuum, and reused for the subsequent run.The final product was recrystallized in ethanol. All products were characterized by NMR, IR, and melting points (the melting points and IR spectra of the compounds were matched well with literature reported data for the corresponding compounds) (Figures S1–S29, ESI).

For 5-(ethoxycarbonyl)-6-methyl-4-phenyl-3,4-dihydropyrimidin-2(1H)-one (entry 1a): Yield 93%, White crystal; mp 201–203 °C; FT-IR (KBr, cm^−1^) ν max 3244, 3115, 2977, 1724, 1647, 1464, 1290, 1220, 1090, 781, 698. ^1^H NMR (DMSO-*d*6): 1.2(3H, t, J = 6.9 Hz, OCH_2_CH_3_), 2.24(3H, s, CH_3_), 3.967 (2H, q, J = 7.2 Hz, OCH_2_CH_3_), 5.136 (d, 1H, J = 3 Hz, –CH), 7.314(m, 5H, Ar–H), 7.68(1H, s, NH), 9.136(1H, s, NH). ^13^C NMR (DMSO-*d*_6_): 14.516, 18.248, 54.436, 59.735, 99.712, 118.537, 126.721, 127.731, 128.855, 144.255, 149.204, 152.645, 165.799.

For 3,4-dihydro-6-methyl-4-(4-nitrophenyl)-5-propionylpyrimidin-2(1H)-one (entry 2a): Yield 89%, Colorless solid; mp 210–212 °C; FT-IR (KBr, cm^−1^) ν max: 3235, 3118, 2976, 1727, 1648, 1610, 1462, 1391, 1214, 1091, 783, 697. ^1^H NMR (DMSO-*d*6): 2.061(3H, t, J = 6.9 Hz, OCH_2_CH_3_), 2.178(3H, s, CH_3_), 2.407(2H, q, J = 7.2 Hz, OCH_2_CH_3_), 5.275(d, 1H, J = 3.3 Hz, −CH), 7.486(2H, d, J = 7.2 Hz, Ar–H), 7.94(1H, s, NH), 8.082(2H, d, J = 8.4 Hz, Ar–H), 9.29(1H, s, NH). ^13^C NMR (DMSO-*d*_6_): 19.604, 31.116, 53.613, 109.925, 124.333, 128.145, 147.128, 149.628, 152.063, 152.505, 194.416.

For 5-(ethoxycarbonyl)-4-(4-chlorophenyl)-6-methyl-3,4-dihydropyrimidin-2(1H)-one (entry 3a): Yield 88%, yellowish powder; mp 213–215 °C; FT-IR (KBr, cm^−1^) ν max: 3242, 3116, 2979, 1723, 1647, 1489, 1291, 1220, 1088, 781, 492. ^1^H NMR (DMSO-*d*_6_): 1.075(3H, t, OCH_2_CH_3_), 2.459(3H, s, CH3), 3.963(2H, q, J = 6.9 Hz, OCH_2_CH_3_), 5.121(d, 1H, J = 2.7 Hz, –CH), 7.118–7.395(4H, m, Ar–H), 7.715(1H, s, NH), 9.193(1H, s, NH). ^13^C NMR (DMSO-*d*_6_): 14.535, 18.278, 53.873, 59.736, 99.261, 128.661, 128.872, 132.253, 144.256, 149.217, 152.421, 165.666.

For 5-(ethoxycarbonyl)-6-methyl-4-(2-hydroxyphenyl)-3,4-dihydropyrimidin-2(1H)-one (entry 4a): Yield 76%, pale yellow powder; mp 214–215 °C; FT-IR (KBr, cm^−1^) ν max: 3342, 3241, 2986, 1667, 1460, 1233, 1091,757.

5-(Ethoxycarbonyl)-4-(Tiophen-2-yl)-6-methyl-3,4-dihydropyrimidin-2(1H)-one (entry 5a): Yield 78%, pale yellow powder; mp 213–215 °C; FT-IR (KBr, cm^−1^) ν max: 3384, 3103, 2924, 1681, 1435, 1228, 1093, 699.

For 5-(ethoxycarbonyl)-4-(4-naphthalene-1-yl)-6-methyl-3,4-dihydropyrimidin-2(1H)-one (entry 6a): Yield 72%, White powder; mp 245–248 °C; FT-IR (KBr, cm^−1^) ν max: 3243, 3117, 2977, 1698, 1646, 1510, 1318, 1280, 1231, 1087, 777.

For 5-(ethoxycarbonyl)-4-(1H-indol-2-yl)-6-methyl-3,4-dihydropyrimidin-2(1H)-one (entry 7a): Yield 91%, red powder; mp 264–266 °C; FT-IR (KBr, cm^−1^) ν max: 3070, 3001, 2802, 2718, 1713, 1583, 1545, 1463, 1429, 1326, 1282, 1213, 1103, 743. ^1^H NMR (DMSO-*d*_6_): 1.2(3H, t, J = 6.9 Hz, OCH_2_CH_3_), 2.24(3H, s, CH_3_), 3.4(2H, q, J = 7.2 Hz, OCH_2_CH_3_), 4.3(d, 1H, J = 3 Hz, –CH), 7.28–8.6(m, 6H, Ar–H and NH), 7.65(1H, s, NH), 12.4(1H, s, NH). ^13^C NMR (DMSO-*d*_6_): 14.557, 61.557, 104.765, 113.673, 115.089, 120.522, 122.703, 124.104, 125.192, 136.928, 138.55, 163.652.

For 5-acetyl-6-methyl-4-phenyl-3,4-dihydropyrimidin-2(1H)-one (entry 1b): Yield 93%, White powder; mp 221–223 °C; FT-IR (KBr, cm^−1^) ν max: 3332, 3223, 1697, 1667, 1414, 1340, 1239, 1094, 698. ^1^H NMR (DMSO-*d*_6_): 2.06(3H, s, CH_3_), 3.32(3H, s, OCH_3_), 5.242(1H, s, −CH), 7.118–7.34(m, 5H, Ar–H), 7.771(1H, s, NH), 9.127(1H, s, NH). ^13^CNMR (DMSO-*d*_6_): 19.412, 30.801, 30.837, 54.312, 110.096, 126.919, 127.842, 129.011, 144.732, 152.749, 194.774.

For 5-methoxycarbonyl-6-methyl-4-(4-nitrophenyl)-3,4-dihydropyrimidin-2(1H)-one (entry 2b): Yield 89%, White powder; mp 233–235 °C; FT-IR (KBr, cm^−1^) ν max: 3368, 3235, 3109, 2946, 1689, 1617, 1348, 1228, 1095, 855, 700.

For 5-methoxycarbonyl-6-methyl-4-(4-chlorophenyl)-3,4-dihydropyrimidin-2(1H)-one (entry 3b): Yield 88%, yellow powder; mp 154–156 °C; FT-IR (KBr, cm^−1^) ν max: 3324, 3219, 3105, 1698, 1675, 1491, 1420, 1342, 1295, 1239, 1093, 938, 700.

For 5-methoxycarbonyl-6-methyl-4-(2-hydroxyphenyl)-3,4-dihydropyrimidin-2(1H)-one (entry 4b): Yield 76%, Pale yellow powder; mp 265–268 °C; FT-IR (KBr, cm^−1^) ν max: 3441, 3351, 3250, 1690, 1660, 1458, 1086, 960, 800, 462.

For 5-methoxycarbonyl-6-methyl-4-(tiophen-2-yl)-3,4-dihydropyrimidin-2(1H)-one (entry 5b): Yield 77%, pale yellow powder; mp 221–223 °C; FT-IR (KBr, cm^−1^) ν max: 3393, 3232, 3102, 1682, 1434, 1232, 1093, 700.

For 5-methoxycarbonyl-6-methyl-4-(4-naphthalene-1-yl)-3,4-dihydropyrimidin-2(1H)-one (entry 6b): Yield 72%, White powder; mp 268–270 °C; FT-IR (KBr, cm^−1^) ν max: 3239, 3100, 1698, 1649, 1431, 1234, 1093, 776.

For 5-methoxycarbonyl-6-methyl-4-(1*H*-indol-2-yl)-3,4-dihydropyrimidin-2(1H)-one (entry 7b): Yield 88%, red powder; mp 264–266 °C; FT-IR (KBr, cm^−1^) ν max: 3294, 3104, 3073, 2995, 2800, 2724, 2630, 1722, 1600, 1464, 1418, 1331, 1222, 1110, 847, 747. ^1^H NMR (DMSO-*d*_6_): 1.18(3H, s, CH_3_), 4.05(3H, s, OCH_3_), 7.5(H, s, –CH), 7.09–7.5(6H, br, Ar–H) and NH), 8.2(1H, s, NH), 8.6(H, s, NH). ^13^C NMR (DMSO-*d*_6_): 52.858, 104.515, 113.795, 115.342, 120.565, 122.984, 124.298, 125.192, 138.661, 163.929.

For 5-acetyl-6-methyl-4-phenyl-3,4-dihydropyrimidin-2(1H)-one (entry 1c): Yield 92%, White powder; mp 231–233 °C; FT-IR (KBr, cm^−1^) ν max: 3268, 1702, 1675, 1599, 1493, 1236, 1106, 767, 704, 571.

For 5-acetyl-6-methyl-4-(4-nitrophenyl)-3,4-dihydropyrimidin-2(1H)-one (entry 2c): Yield 89%, White powder; mp 229–230 °C; FT-IR (KBr, cm^−1^) ν max: 3342, 3252, 3143, 1709, 1674, 1608, 1515, 1446, 1384, 1239, 1279, 1237, 1187, 1102, 862, 763, 698.

For 5-acetyl-6-methyl-4-(4-chlorophenyl)-3,4-dihydropyrimidin-2(1H)-one (entry 3c): Yield 88%, yellow powder; mp 204–206 °C; FT-IR (KBr, cm^−1^) ν max: 3288, 3121, 2915, 1699, 1618, 1424, 1322, 1262, 1236, 1091, 837, 789, 581.

For 5-acetyl-6-methyl-4-(2-hydroxyphenyl)-3,4-dihydropyrimidin-2(1H)-one (entry 4c): Yield 76%, Pale yellow powder; mp 204–208 °C; FT-IR (KBr, cm^−1^) ν max: 3240, 3096, 2982, 1682, 1603, 1584, 1503, 1173, 1113, 925, 867, 762.

For 5-acetyl-6-methyl-4-(tiophen-2-yl)-3,4-dihydropyrimidin-2(1H)-one (entry 5c): Yield 74%, pale yellow powder; mp 231–233 °C; FT-IR (KBr, cm^−1^) ν max: 3550, 3473, 3413, 1680, 1617, 1236, 698, 617.

For 5-acetyl-6-methyl-4-(4-Naphthalene-1-yl)-3,4-dihydropyrimidin-2(1H)-one (entry 6c): Yield 70%, White powder; mp 233–236 °C; FT-IR (KBr, cm^−1^) ν max: 3328, 3212, 3110, 2917, 1693, 1607, 1415, 1381, 1321, 1230, 772.

For 4-(1-H-indole-2-yl)-3,4-dihydropyrimidin-2(1H)-one (entry 7c): Yield 90%, red powder; mp 128–130 °C; FT-IR (KBr, cm^−1^) ν max: 3439, 3109, 2933, 1762, 1598, 1505, 1472, 1433, 1328, 1234, 1129, 1055, 798, 749. ^1^H NMR (DMSO-*d*_6_): 1.95(s, 3H, CH_3_), 2.377(s, 3H, COCH_3_), 5.455(d, 1H, CH), 6.65 (s, 1H, NH), 6(m, 4H, ArH) and NH, 8.153(s, 1H, NH) 8.153(s, 1H, NH). ^13^C NMR (DMSO-*d*_6_): 112.901, 114.35, 118.597, 121.271, 122.581, 123.914, 124.561, 125,124, 137.512, 138.61, 138.965, 141,628, 160.304, 160.512, 185.449.

## 3. Results and Discussion

**Scheme 1:**
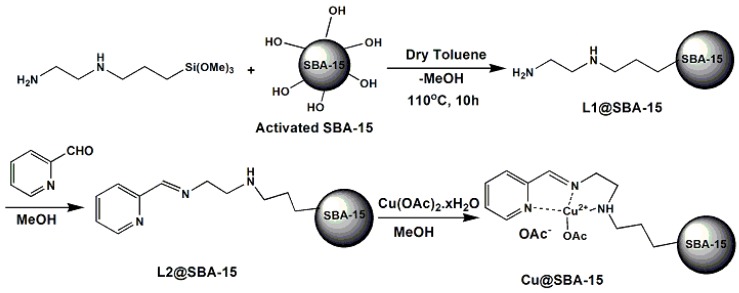
Synthesis of supported copper Schiff base tridentate complex nanocatalyst.

We have anchored a copper Schiff base tridentate complex immobilized on SBA-15 nanoreactors using an easy 3-step system (Scheme 1) that takes advantage of the biologically active properties and coordination chemistry of copper.

**Figure 2:**
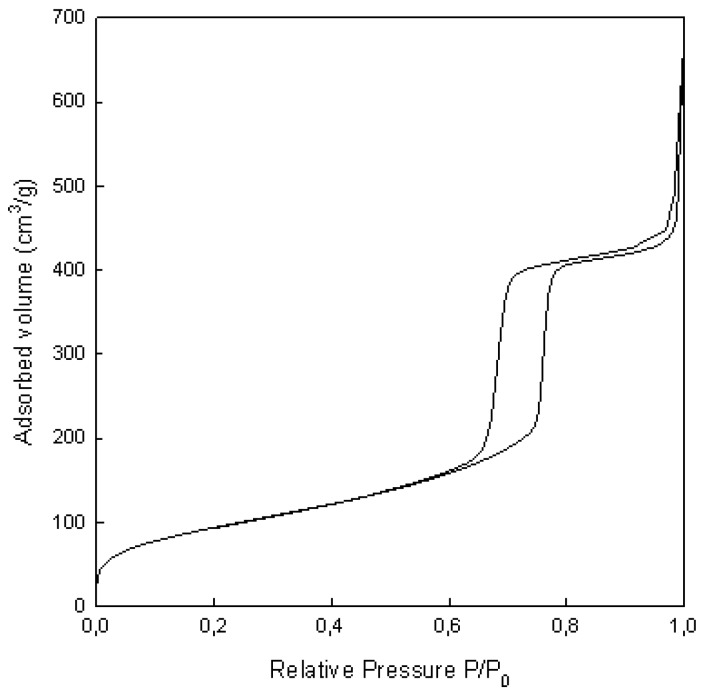
N_2_ adsorption-desorption isotherm of the Cu@SBA-15 nanocatalyst.

Brunauer-Emmett-Teller (BET) surface area, calculated from the adsorption/desorption isotherm and pore size distribution of Cu@SBA-15, is presented in Figure 2. Cu@SBA-15 shows a type IV isotherm with a hysteresis typical for a mesoporous material possessing a pore diameter between 2 nm and 50 nm.

**Figure 3:**
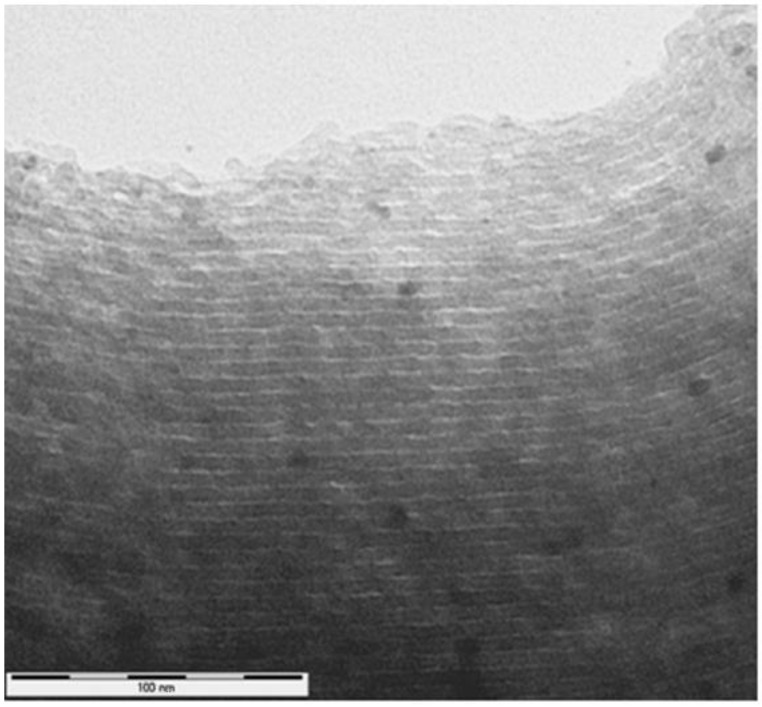
TEM image of the Cu@SBA-15 nanocatalyst.

A narrow pore size distribution, calculated from desorption isotherms with the Barrett-Joyner-Halenda (BJH) method, indicates the uniformity of the mesopores in the Cu@SBA-15 material. The measured data for the BET surface area and the total pore volume and the BJH pore size of Cu@SBA-15 are 346 m^2^g^−1^ and 0.61 cm^3^g^−1^, respectively, with a mean pore diameter 8.6 nm. Furthermore, the highly ordered mesostructure of the resulting material was confirmed by TEM measurements (Figure 3).

**Scheme 2:**
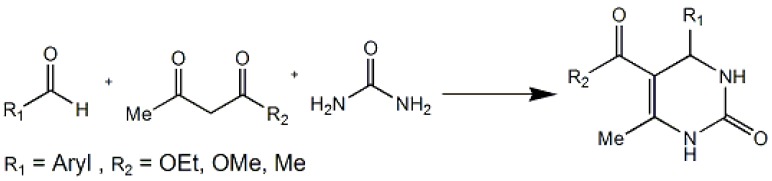
Three-component coupling Biginelli reaction of β-dicarbonyl compounds, aldehyde, and urea.

The catalytic activity of the supported Cu(II) nanocatalyst has been investigated in the reaction of three-component coupling of ethyl acetoacetate, benzaldehyde, and urea as a model reaction (Scheme 2, R_1_ = Ph, R_2_ = OEt). The effects of reaction temperature, catalyst amount, and solvent were examined to optimize the reaction parameters (Table 1). The reaction was performed in the absence of supported Cu(II) nanocatalyst, and poor results were obtained after 3 h at 100 °C (under 20% yield, Table 1, entry 1). Initially, different amounts of supported Cu(II) nanocatalyst were studied at 100 °C under solventless conditions, and the product yields increased from 48% to 94% by increasing the catalyst loading from 4 mg to 10 mg (Table 1, entries 2–5). However, no changes in yields were observed when the amount of increased to 12 mg (Table 1, entry 6). The reaction was then carried out in the presence of 10 mg supported Cu(II) nanocatalyst at different temperatures. Reaction yields generally decreased, as expected, with a temperature decrease (Table 1, entries 8–10). Polar aprotic and protic solvents such as EtOH, DMF, CH_3_CN, THF, and CHCl_3_ resulted in low to moderate yields of the product under reflux conditions after 10 min (Table 1, entries 12–16). Notably, it was observed that the reaction gave the highest yield of product after 5 min in the presence of 10 mg (0.4 mol%) of the supported Cu(II) nanocatalyst under solventless conditions (Table 1, entry 5). These were considered as optimum conditions for this work.

Having established the reaction conditions, the scope and limitations of the process were further investigated using different aldehydes, β-dicarbonyl compounds, and urea (Table 2). Cu@SBA-15 provided excellent yields for different substrates in all reactions. A variety of aromatic aldehydes bearing electron donating and withdrawing groups gave corresponding 3,4-dihydropyrimidin-2(1H)-ones in moderate to high yields through the reaction with *β*-dicarbonyl compounds and urea using 0.4 mol% of supported nanocatalyst under solvent free conditions. The results are shown in Table 2. In all cases, dihydropyrimidinones were the sole products, and no collateral compounds were observed (Table 2, entry 1a–7c). Various aromatic aldehydes, including benzaldehyde, 4-nitro-, 4-chloro-, 2-hydroxy- benzaldehyde, thiophene-2-carbaldehyde, 1-naphthaldehyde, and indole-3-carbaldehyde using equivalents of ethylacetoacetate, methyl acetoacetate, and acetyl acetone and an excess amount of urea and 10 mg of Cu catalyst, afforded products 1a–7c in good to excellent yields (70–94%).

**Figure 4:**
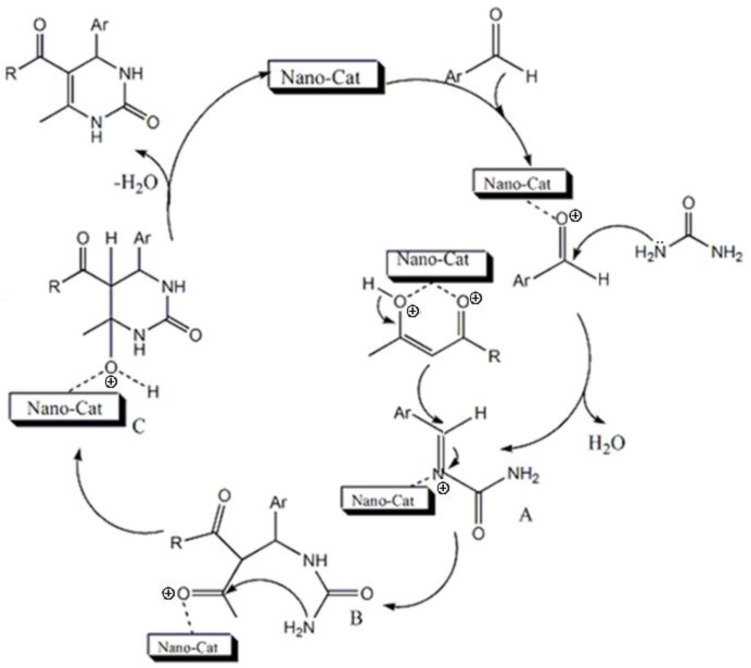
Proposed mechanism for one-pot synthesis of dihydropyrimidinones derivatives from aldehyde, ethyl acetoacetate, and urea using Cu@SBA-15.

It seems that the presence of electron-withdrawing or electron-donating groups and their position on the aromatic ring of the aldehydes does not significantly influence reaction yields in dihydropyrimidinones synthesis. Based on the mechanism suggested by Kappe [2], a proposed mechanism for the formation of DHPMs is presented in Figure 4.

The first step in the mechanism is believed to be the activation of aldehyde by the catalyst following condensation by urea forming the A intermediate. The next step is the formation of B intermediate in nucleophilic addition of *β*-dicarbonyl compounds onto the A intermediate. The reaction subsequently proceeds via cyclization to the C intermediate by elimination of water to afford dihydroprimidin-2(1H)-one.

Recyclability is an important feature that determines the stability and reutilization of heterogeneous catalysts in multiple subsequent runs. We studied the recyclability of Cu@SBA-15 in the optimized model reaction. Upon completion of the first Biginelli reaction under optimized conditions, Cu@SBA-15 was separated by simple filtration from the reaction mixture, washed with water and ethanol, dried in vacuum, and reused for the next reaction cycle. The recovered catalyst still shows remarkable activity for ten subsequent reactions (>90% conversion) under the same conditions as the fresh catalyst and exhibited constant catalytic activity, indicating the outstanding reusability of this heterogeneous catalyst.

Table 3 show a comparison of the efficiency of our nanocatalyst with others reported in the literature for the synthesis of DHPMs. As can be seen in Table 3, our recoverable catalytic system possesses good activity as compared to those of previously reported heterogeneous catalytic systems.

## 4. Conclusions

In summary, we have developed an eco-friendly and highly efficient copper tridentate Schiff base complex on mesoporous SBA-15 material (Cu@SBA-15) for the one-pot Biginelli reaction coupling of β-dicarbonyl compounds as a source of two carbon fragments, aromatic aldehydes, and urea to afford the corresponding dihydropyrimidinones (DHPMs) under solvent free conditions at 100 °C. The supported copper nanocatalyst displayed a notable stability under these conditions and could be easily separated from the reaction mixture by simple filtration. The catalyst could be easily recycled for ten reaction runs without any major activity loss. This novel synthetic method has several advantages, including high yields, cost-effectiveness, practical simplicity, high selectivity, low catalyst loading, and easy work-up. Further work is under investigation in our laboratory on the use of supported copper nanocatalyst in other reactions, such as condensation, couplings, and oxidation reactions.

## Figures and Tables

**Table 1:** Effects of different parameters on the Biginelli reaction of ethyl acetoacetate (5 mmol), benzaldehyde (5 mmol), urea (6 mmol).

Entry	Cu@SBA-15 (mg)	Solvent	Temp. (°C)	Yield (%)^a^
1	-	-	100	<20
2	4	-	100	48
3	6	-	100	78
4	8	-	100	87
5	10	-	100	94
6	12	-	100	94
8	10	-	25	25
9	10	-	60	41
10	10	-	80	78
12	10	EtOH	Reflux	73
13	10	CHCl_3_	Reflux	35
14	10	THF	Reflux	38
15	10	DMF	Reflux	75
16	10	CH_3_CN	Reflux	65

^a^ Isolated yield.

**Table 2:** Synthesis of dihydropyrimidinones catalyzed by Cu@SBA-15 under solvent free conditions ^a^.

Entry	Aldehyde	β-Keto Ester	Time (min)	Yield (%) ^b^	Mp (°C)	Ref
1a	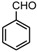	Ethyl acetoacetate	5	94	201–203	[19]
2a	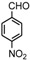	Ethyl acetoacetate	5	91	211–213	[20]
3a	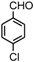	Ethyl acetoacetate	5	92	210–212	[21]
4a	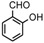	Ethyl acetoacetate	5	82	217–219	[22]
5a	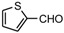	Ethyl acetoacetate	10	75	215–217	[23]
6a	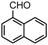	Ethyl acetoacetate	6	77	245–247	[24]
7a	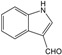	Ethyl acetoacetate	5	89	264–267	New
1b	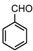	Methyl acetoacetate	5	90	221–223	[25]
2b	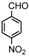	Methyl acetoacetate	5	88	233–235	[26]
3b	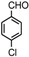	Methyl acetoacetate	5	85	154–156	[27]
4b	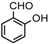	Methyl acetoacetate	5	72	243–244	[28]
5b	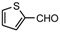	Methyl acetoacetate	10	78	221–222	[29]
6b	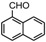	Methyl acetoacetate	10	70	221–223	[30]
7b	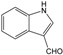	Methyl acetoacetate	5	89	268–270	New
1c	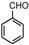	Acethyl acetone	5	91	231–233	[31]
2c	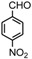	Acethyl acetone	5	88	229–230	[32]
3c	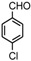	Acethyl acetone	5	87	204–206	[33]
4c	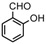	Acethyl acetone	5	73	215–217	[34]
5c	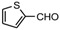	Acethyl acetone	10	71	231–233	[35]
6c	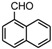	Acethyl acetone	10	70	233–236	-
7c	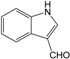	Acethyl acetone	5	90	128–130	-

^a^ Reaction conditions: aldehyde (5 mmol), β-dicarbonyl (5 mmol), urea (6 mmol), and Cu@SBA-15 (10 mg, 0.02 mmol), 100 °C; ^b^ Isolated yield.

**Table 3:** Comparison of the efficiency of Cu@SBA-15 in the reaction of benzaldehyde, α-dicarbonyl compounds, and urea, and the previous literature.

Entry	condition/Catalyst	Time	Conversion (%)	Ref.
1	Cu@SBA-15	5 min	94	This work
2	TiCl_4_/Solvent free/80 °C	60 s	75	[36]
3	TSILS (ionic liquids)/90 °C	10 min	94	[37]
4	CuS QD/MW	120 min	97	[38]
5	Zn-MOF/Solvent free/80 °C	110 min	94	[39]
6	ALKIT-5(10)/CH_3_CN/Reflux	180 min	96	[40]
7	β-Cyclodexterin (0.5 mol%)/Solvent free/100 °C	180 min	85	[41]
8	Montmorillonite KSF/Toluene/100 °C	48 h	82	[42]
9	10 mol% Acid/1,4-dioxane/CHCl_3_(8:2)/25 °C	3 days	96	[43]

## Supplementary Materials

The following are available online at http://www.mdpi.com/1996-1944/11/12/2458/s1, Figure S1: ^1^H NMR and ^13^C NMR spectra of 5-(ethoxycarbonyl)-6-methyl-4-phenyl-3,4-dihydropyrimidin-2(1H)-one, Figure S2: ^1^H NMR and ^13^C NMR spectra of 5-(ethoxycarbonyl)-6-methyl-4-(4-nitrophenyl)-3,4-dihydropyrimidin-2(1H)-one, Figure S3: ^1^H NMR and ^13^C NMR spectra of 3,4-dihydro-6-methyl-4-(4-nitrophenyl)-5-propionylpyrimidin-2(1H)-one, Figure S4: ^1^H NMR and ^13^C NMR spectra of 5-(methoxycarbonyl)-6-methyl-4-phenyl-3,4-dihydropyrimidin-2(1H)-one, Figure S5: ^1^H NMR and ^13^C NMR spectra of 5-(ethoxycarbonyl)-4-(1*H*-indol-2-yl)-6-methyl-3,4-dihydropyrimidin-2(1H)-one, Figure S6: ^1^H NMR and ^13^C NMR spectra of 5-(methoxycarbonyl)-4-(1*H*-indol-2-yl)-6-methyl-3,4-dihydropyrimidin-2(1H)-one, Figure S7: ^1^H NMR and ^13^C NMR spectra of 5-acetyl-6-methyl-4-(1-H-indole-2-yl)-3,4-dihydropyrimidin-2(1H)-one, Figure S8: FTIR of 5-(methoxycarbonyl)-6-methyl-4-phenyl-3,4-dihydropyrimidin-2(1H)-one, Figure S9: FTIR of 5-(methoxycarbonyl)-4-(1*H*-indol-2-yl)-6-methyl-3,4-dihydropyrimidin-2(1H)-one, Figure S10: FTIR of 5-(ethoxycarbonyl)-4-(tiophen-2-yl)-6-methyl-3,4 dihydropyrimidin-2(1H)-one, Figure S11: FTIR of 5-(ethoxycarbonyl)-6-methyl-4-phenyl-3,4-dihydropyrimidin-2(1H)-one, Figure S12: FTIR of 5-acetyl-6-methyl-4-(tiophen-2-yl)-3,4-dihydropyrimidin-2(1H)-one, Figure S13: FTIR of 5-acetyl-6-methyl-4-(1-H-indole-2-yl)-3,4-dihydropyrimidin-2(1H)-one, Figure S14: FTIR of 5-(ethoxycarbonyl)-4-(1*H*-indol-2-yl)-6-methyl-3,4-dihydropyrimidin-2(1H)-one, Figure S15: FTIR of 5-acetyl-6-methyl-4-(4-nitrophenyl)-3,4-dihydropyrimidin-2(1H)-one, Figure S16: FTIR of 5-(ethoxycarbonyl)-6-methyl-4-(4-nitrophenyl)-3,4-dihydropyrimidin-2(1H)-one, Figure S17: FTIR of 5-methoxycarbonyl-6-methyl-4-(tiophen-2-yl)-3,4-dihydropyrimidin-2(1H)-one, Figure S18: FTIR of 5-(ethoxycarbonyl)-6-methyl-4-(2-hydroxyphenyl)-3,4-dihydropyrimidin-2(1H)-one, Figure S19: FTIR of 5-(methoxycarbonyl)-6-methyl-4-(4-nitrophenyl)-3,4-dihydropyrimidin-2(1H)-one, Figure S20: FTIR of 5-acetyl-6-methyl-4-(4-chlorophenyl)-3,4-dihydropyrimidin-2(1H)-one, Figure S21: FTIR of 5-(ethoxycarbonyl)-4-(4-chlorophenyl)-6-methyl-3,4-dihydropyrimidin-2(1H)-one, Figure S22: FTIR of 5-(methoxycarbonyl)-4-(4-chlorophenyl)-6-methyl-3,4-dihydropyrimidin-2(1H)-one, Figure S23: FTIR of 5-(ethoxycarbonyl)-4-(4-naphthalene-1-yl)-6-methyl-3,4-dihydropyrimidin-2(1H)-one, Figure S24: FTIR of 5-(methoxycarbonyl)-6-methyl-4-(2-hydroxyphenyl)-3,4-dihydropyrimidin-2(1H)-one, Figure S25: FTIR of 5-acetyl-6-methyl-4-(4-naphthalene-1-yl)-3,4-dihydropyrimidin-2(1H)-one, Figure S26: FTIR of 5-acetyl-6-methyl-4-(2-hydroxyphenyl)-3,4-dihydropyrimidin-2(1H)-one, Figure S27: FTIR of 5-methoxycarbonyl-6-methyl-4-(4-naphthalene-1-yl)-3,4-dihydropyrimidin-2(1H)-one, Figure S28: ^29^Si NMR of Cu@SBA-15 nanostructures, Figure S29: ^13^C NMR of Cu@SBA-15 nanostructures.
