# Supplementary material for: Copper Tridentate Schiff Base Complex Supported on SBA-15 as Efficient Nanocatalyst for Three-Component Reactions under Solventless Conditions
Source: Materials (Basel). 2018 Dec 4;11(12):2458. doi: 10.3390/ma11122458 (PMC6316914; doi:10.3390/ma11122458)
Supplement: Supplementary file 1 [file materials-11-02458-s001.pdf]

Supplementary Information

¹H NMR, ¹³C NMR, and FTIR spectra

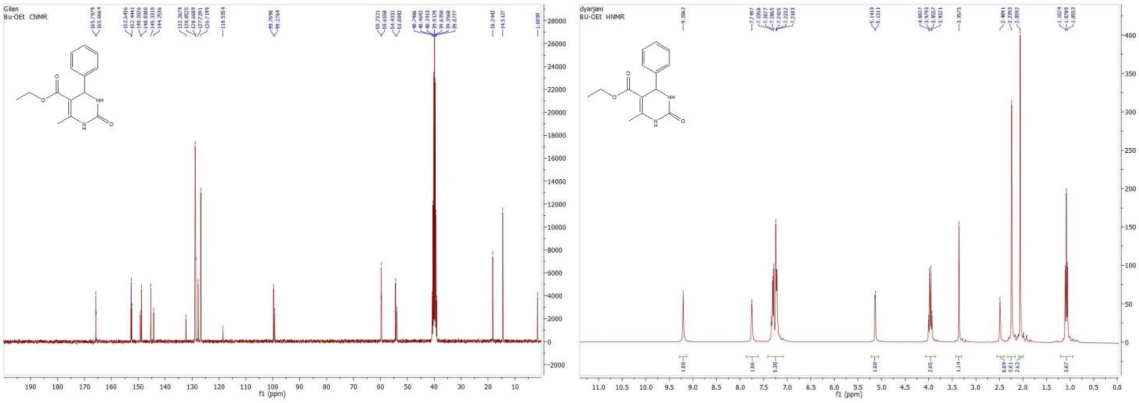

Figure S1. ¹H NMR and ¹³C NMR spectra of 5-(ethoxycarbonyl)-6-methyl-4-phenyl-3,4-dihydropyrimidin-2(1H)-one.

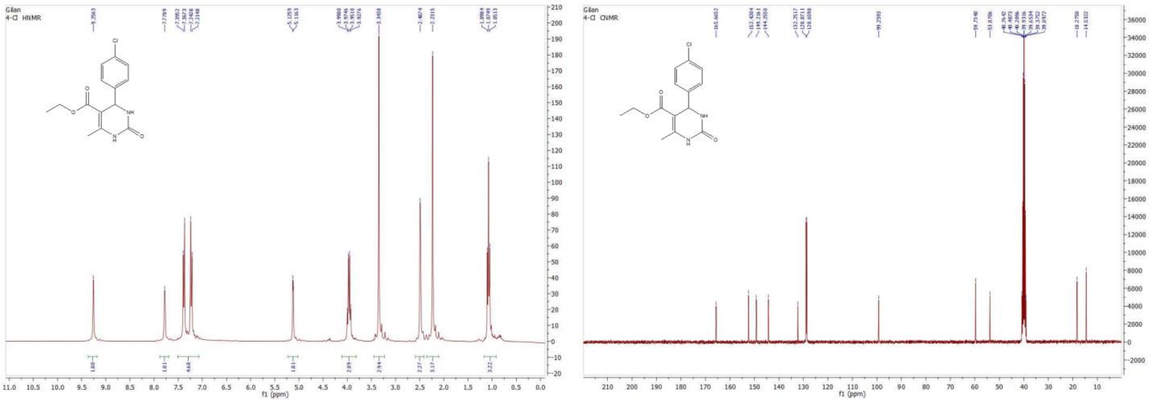

Figure S2. ¹H NMR and ¹³C NMR spectra of 5-(ethoxycarbonyl)-6-methyl-4-(4-nitrophenyl)-3,4-dihydropyrimidin-2(1H)-one.

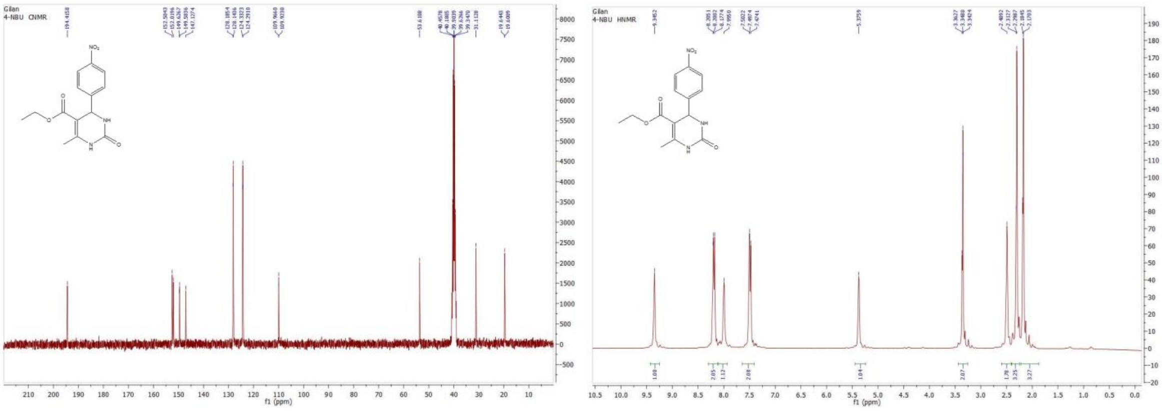

Figure S3. ¹H NMR and ¹³C NMR spectra of 3,4-dihydro-6-methyl-4-(4-nitrophenyl)-5-propionylpyrimidin-2(1H)-one.

Materials **2018**, *11*, 2458; doi:10.3390/ma11122458 www.mdpi.com/journal/materials

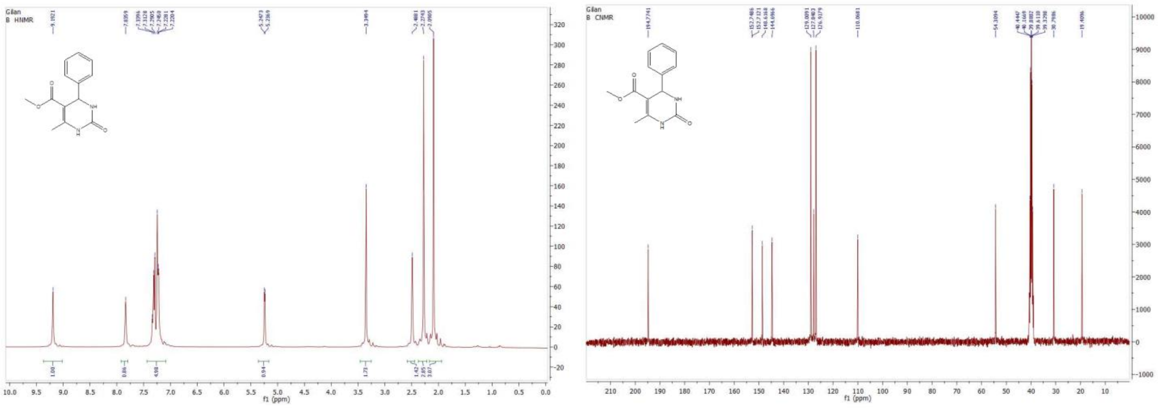

Figure S4. ^{1}H NMR and ^{13}C NMR spectra of 5-(methoxycarbonyl)-6-methyl-4-phenyl-3,4-dihydropyrimidin-2(1H)-one.

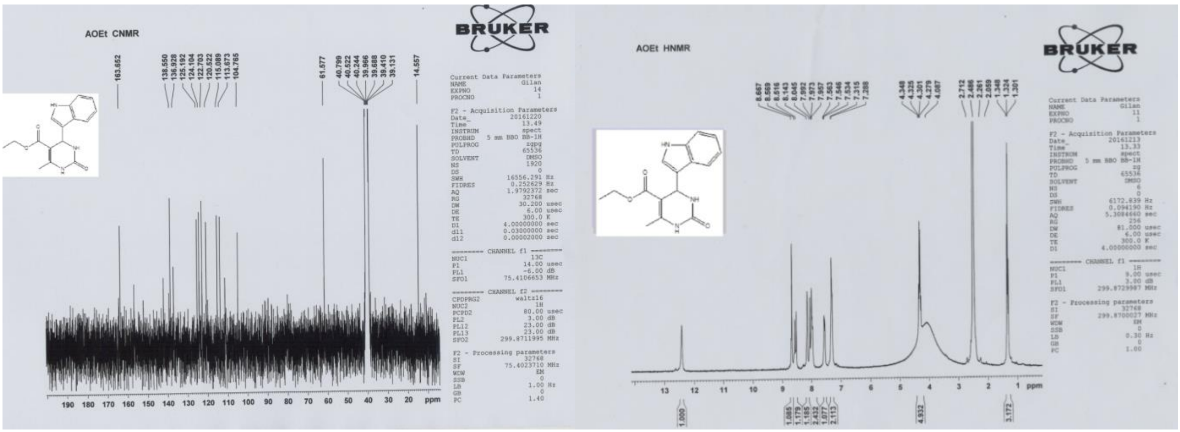

Figure S5. ^{1}H NMR and ^{13}C NMR spectra of 5-(ethoxycarbonyl)-4-(1*H*-indol-2-yl)-6-methyl-3,4-dihydropyrimidin-2(1H)-one.

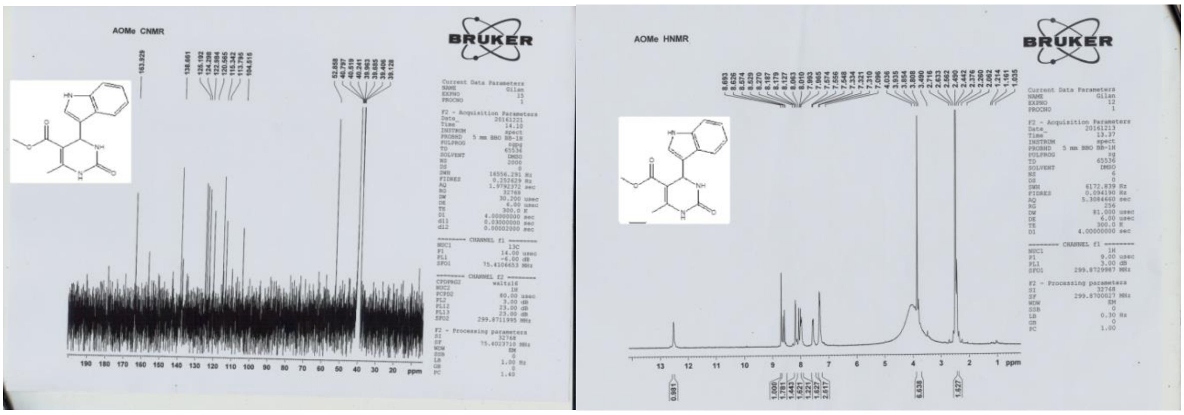

Figure S6. ^{1}H NMR and ^{13}C NMR spectra of 5-(methoxycarbonyl)-4-(1*H*-indol-2-yl)-6-methyl-3,4-dihydropyrimidin-2(1H)-one.

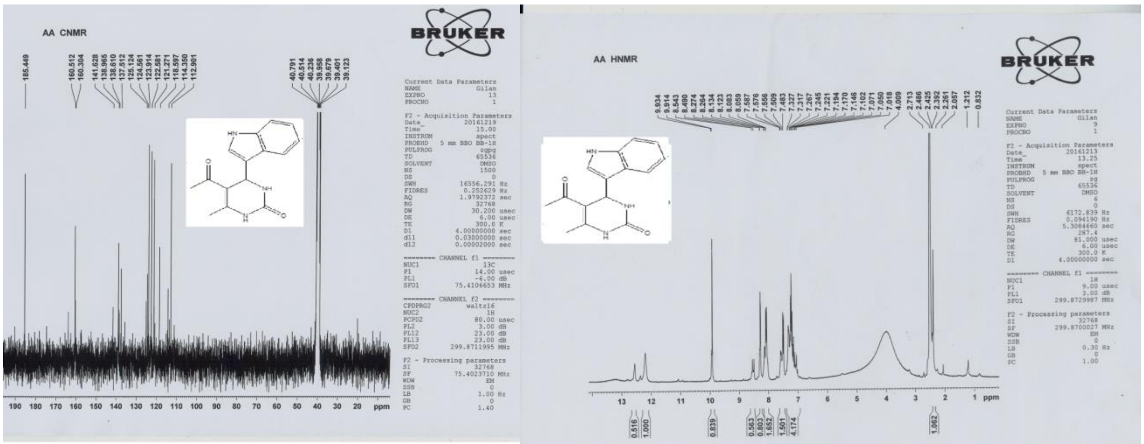

Figure S7. ^{1}H NMR and ^{13}C NMR spectra of 5-acetyl-6-methyl-4-(1-H-indole-2-yl)-3,4-dihydropyrimidin-2(1H)-one.

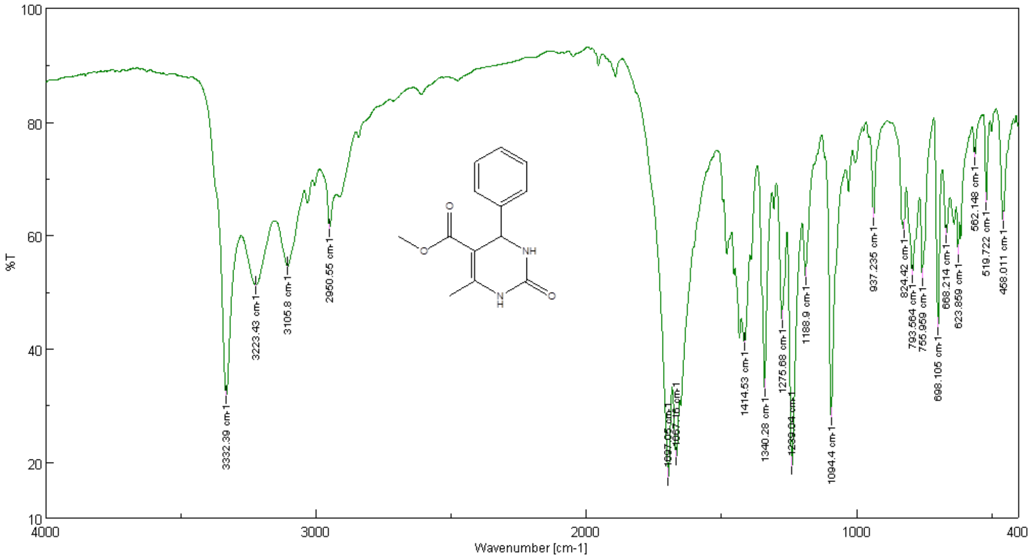

Figure S8. FTIR of 5-(methoxycarbonyl)-6-methyl-4-phenyl-3,4-dihydropyrimidin-2(1H)-one.

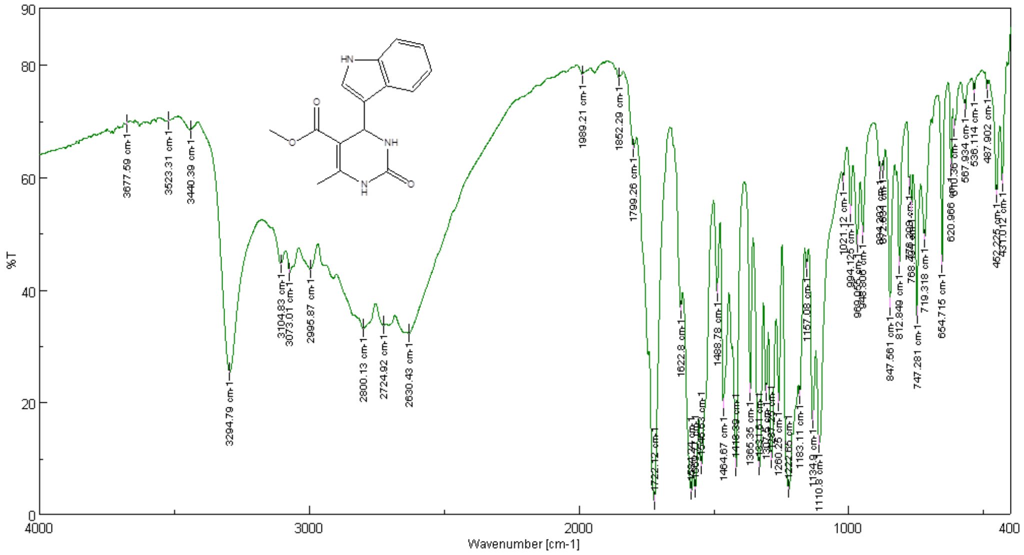

Figure S9. FTIR of 5-(methoxycarbonyl)-4-(1*H*-indol-2-yl)-6-methyl-3,4-dihydropyrimidin-2(1H)-one.

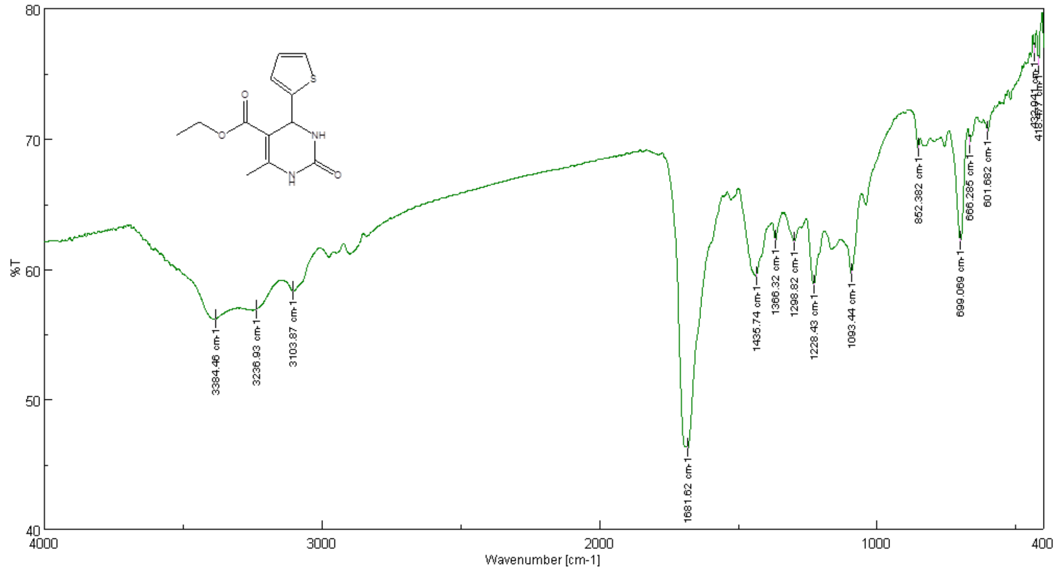

Figure S10. FTIR of 5-(ethoxycarbonyl)-4-(tiophen-2-yl)-6-methyl-3,4 dihydropyrimidin-2(1H)-one.

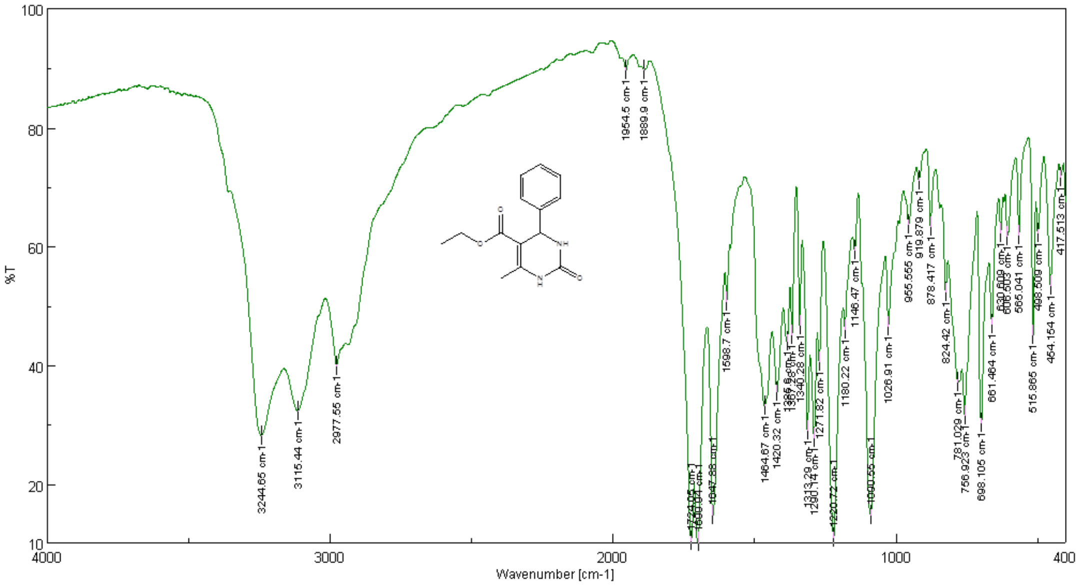

Figure S11. FTIR of 5-(ethoxycarbonyl)-6-methyl-4-phenyl-3,4-dihydropyrimidin-2(1H)-one.

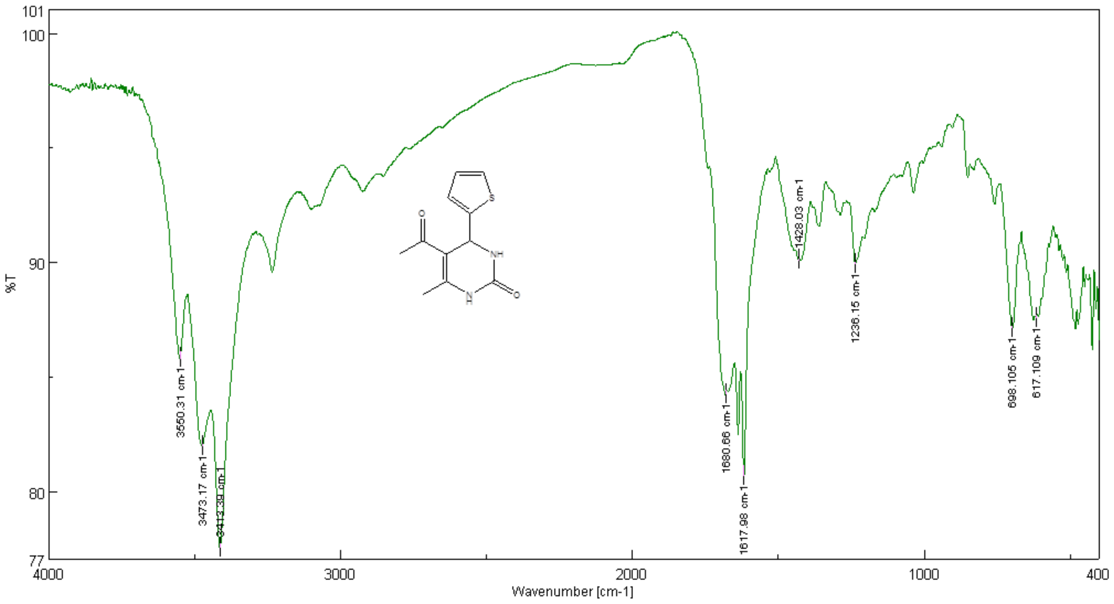

Figure S12. FTIR of 5-acetyl-6-methyl-4-(tiophen-2-yl)-3,4-dihydropyrimidin-2(1H)-one.

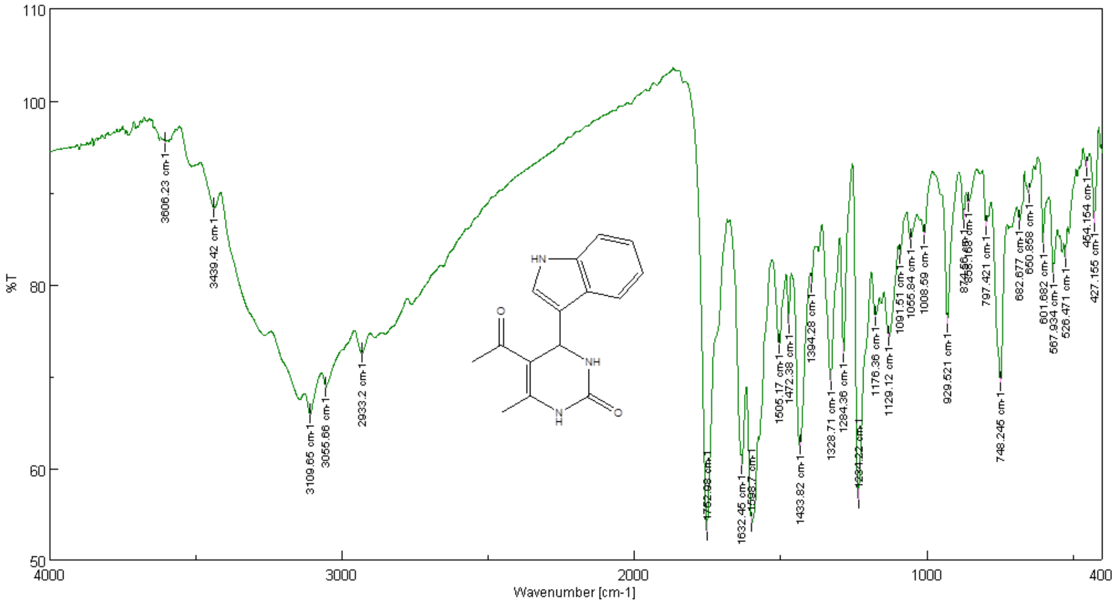

Figure S13. FTIR of 5-acetyl-6-methyl-4-(1-H-indole-2-yl)-3,4-dihydropyrimidin-2(1H)-one.

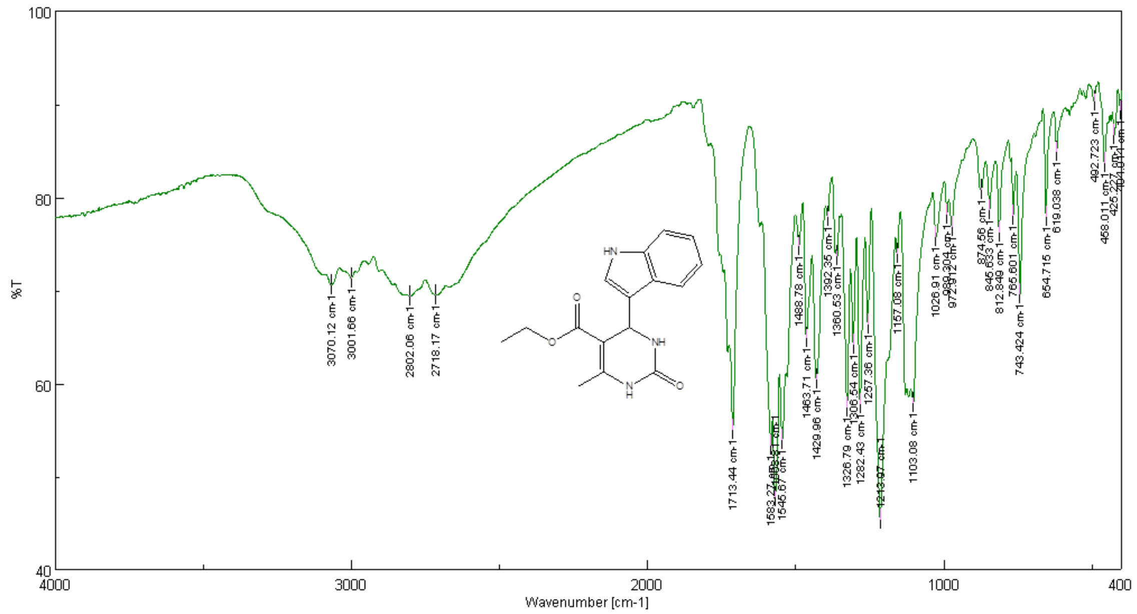

Figure S14. FTIR of 5-(ethoxycarbonyl)-4-(1*H*-indol-2-yl)-6-methyl-3,4-dihydropyrimidin-2(1H)-one.

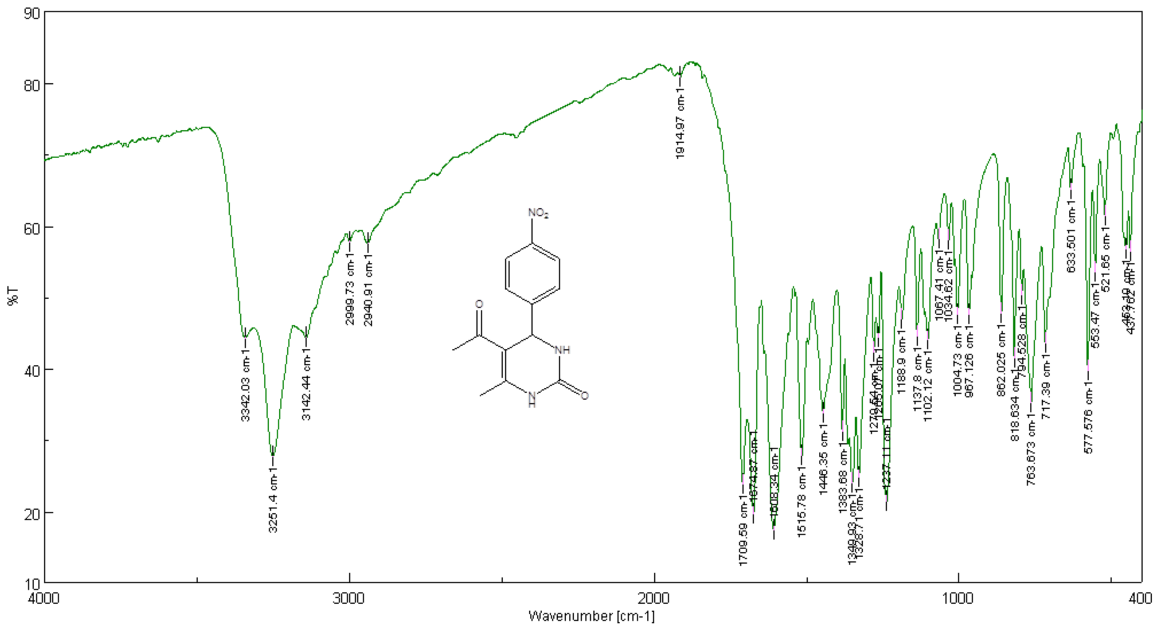

Figure S15. FTIR of 5-acetyl-6-methyl-4-(4-nitrophenyl)-3,4-dihydropyrimidin-2(1H)-one.

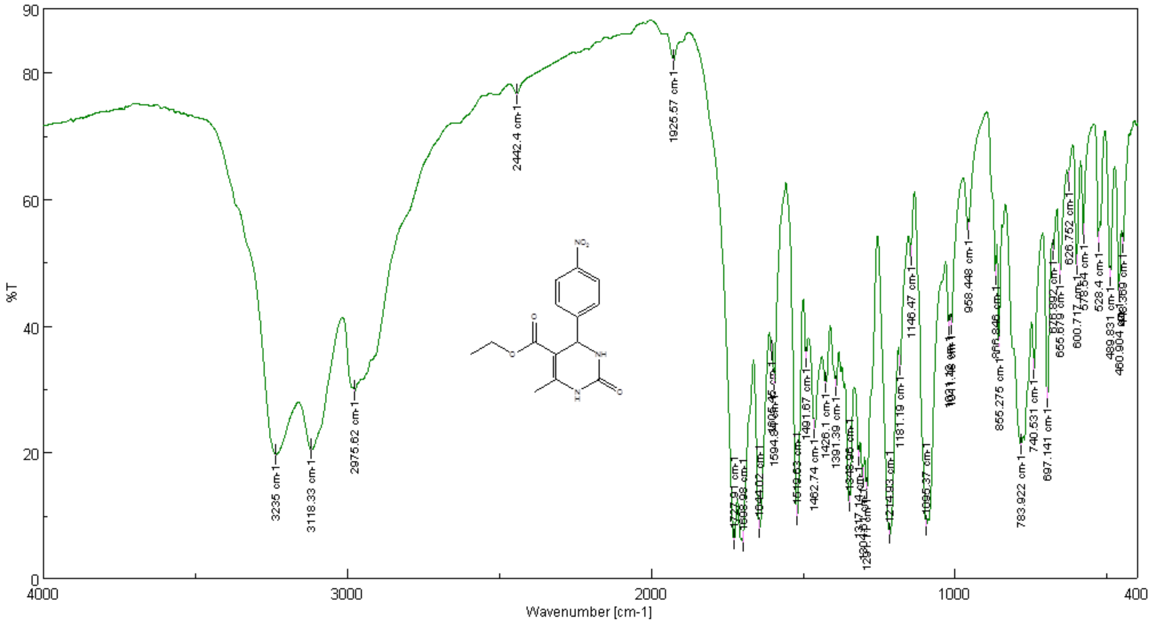

Figure S16. FTIR of 5-(ethoxycarbonyl)-6-methyl-4-(4-nitrophenyl)-3,4-dihydropyrimidin-2(1H)-one.

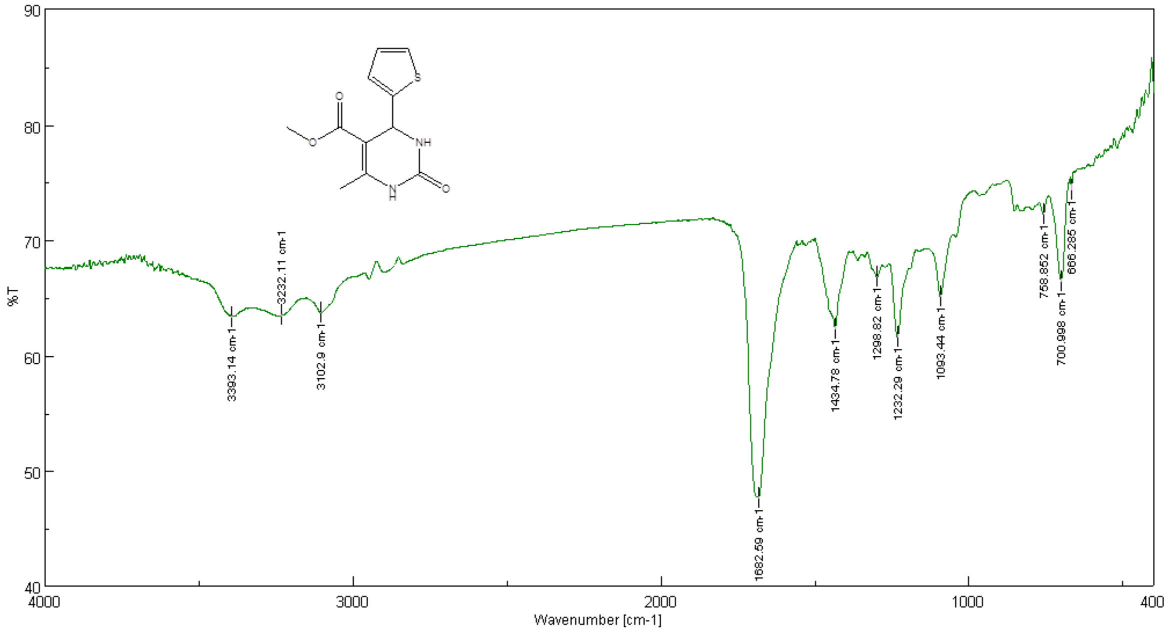

Figure S17. FTIR of 5-methoxycarbonyl-6-methyl-4-(tiophen-2-yl)-3,4-dihydropyrimidin-2(1H)-one.

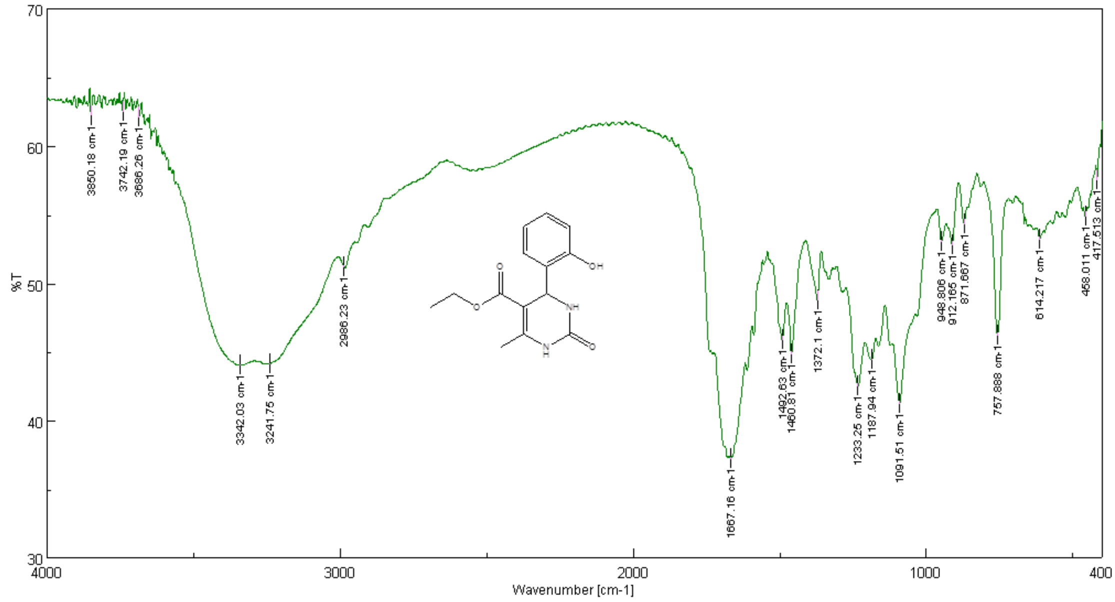

Figure S18. FTIR of 5-(ethoxycarbonyl)-6-methyl-4-(2-hydroxyphenyl)-3,4-dihydropyrimidin-2(1H)-one.

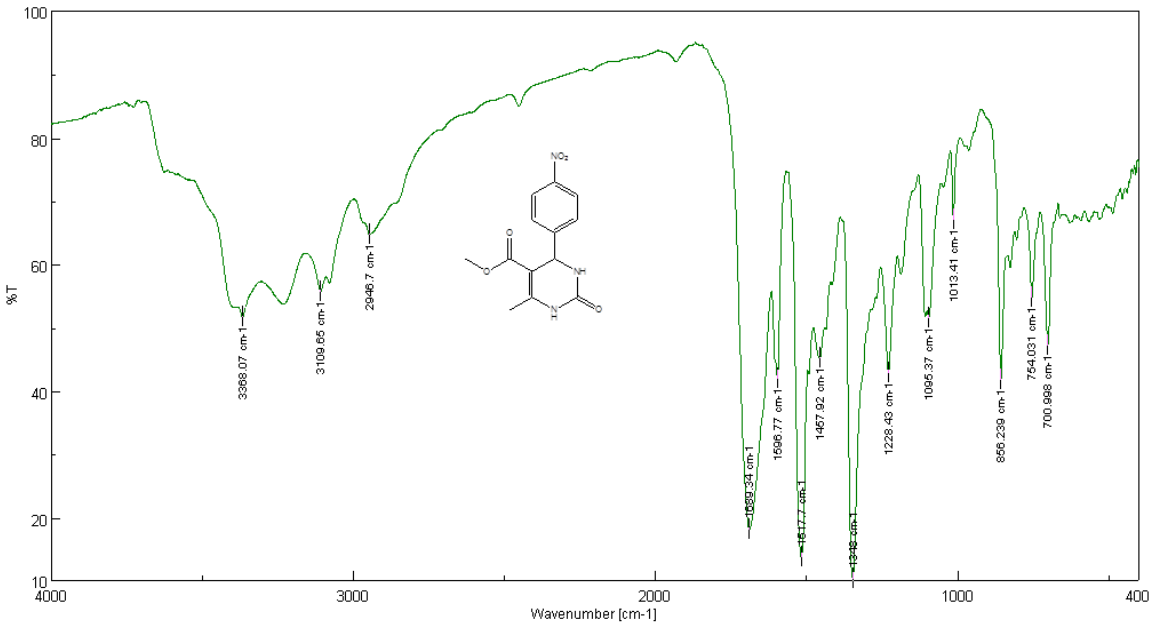

FigureS19. FTIR of 5-(methoxycarbonyl)-6-methyl-4-(4-nitrophenyl)-3,4-dihydropyrimidin-2(1H)-one.

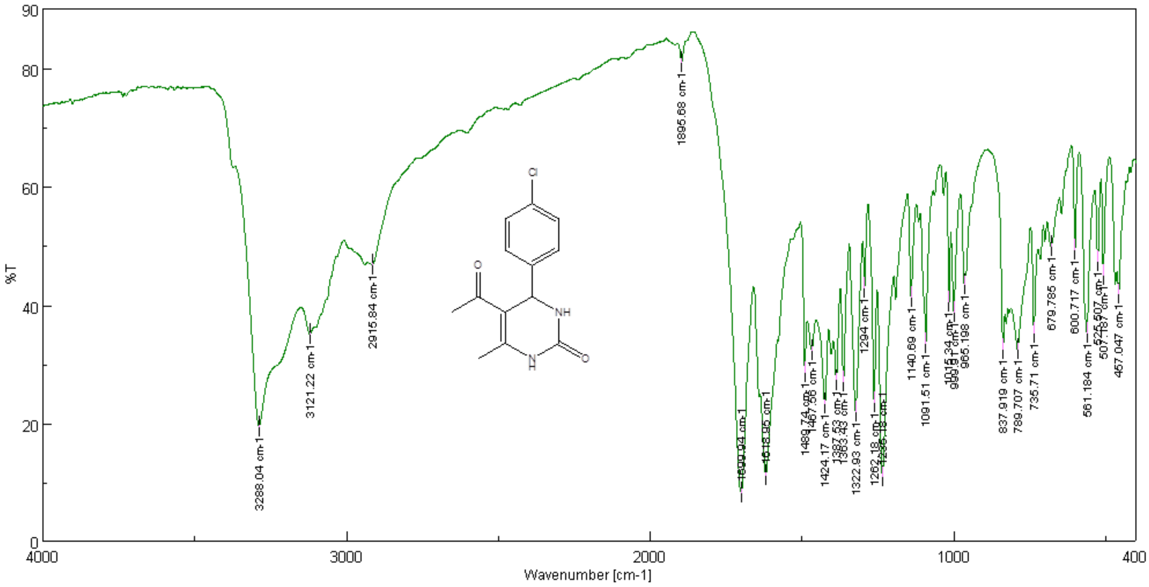

Figure S20. FTIR of 5-acetyl-6-methyl-4-(4-chlorophenyl)-3,4-dihydropyrimidin-2(1H)-one.

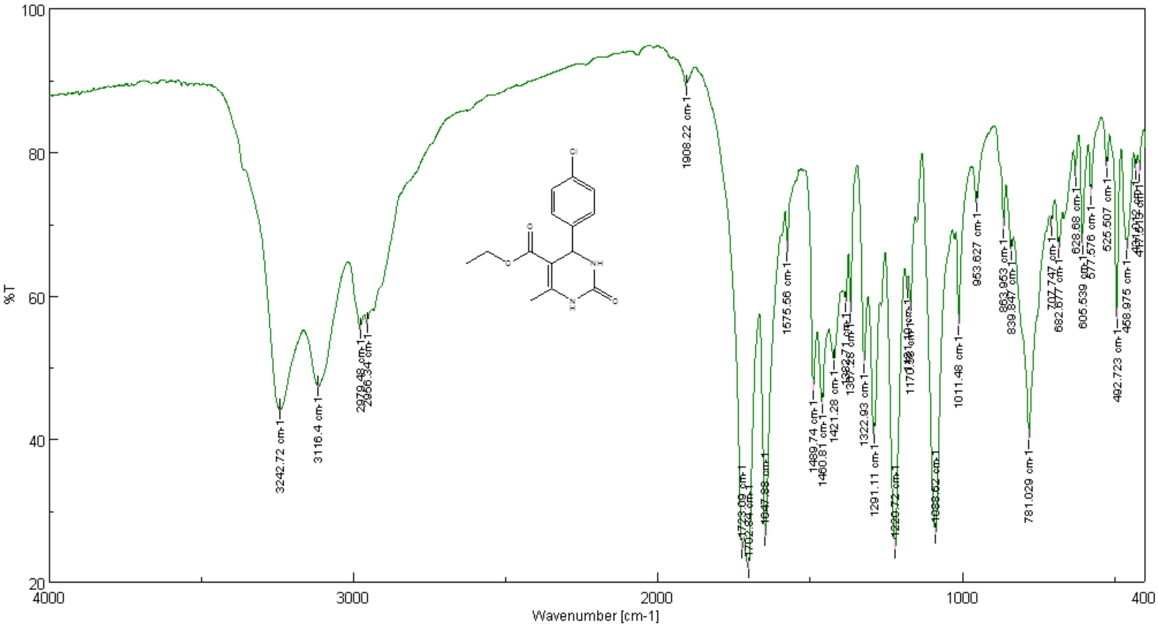

FigureS21. FTIR of 5-(ethoxycarbonyl)-4-(4-chlorophenyl)-6-methyl-3,4-dihydropyrimidin-2(1H)-one.

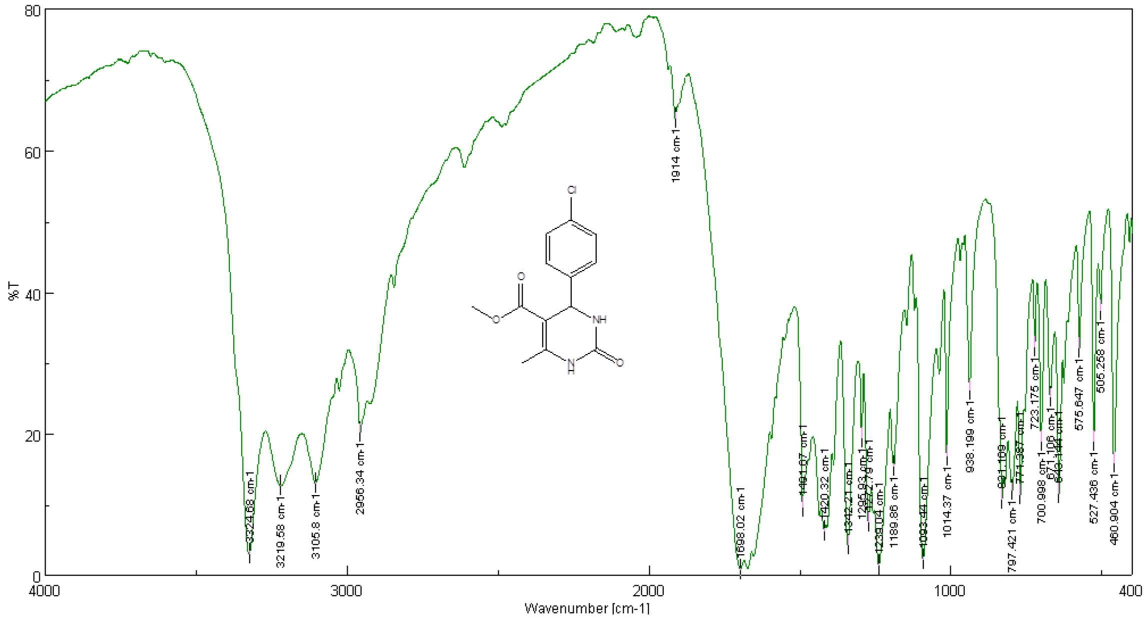

Figure S22. FTIR of 5-(methoxycarbonyl)-4-(4-chlorophenyl)-6-methyl-3,4-dihydropyrimidin-2(1H)-one.

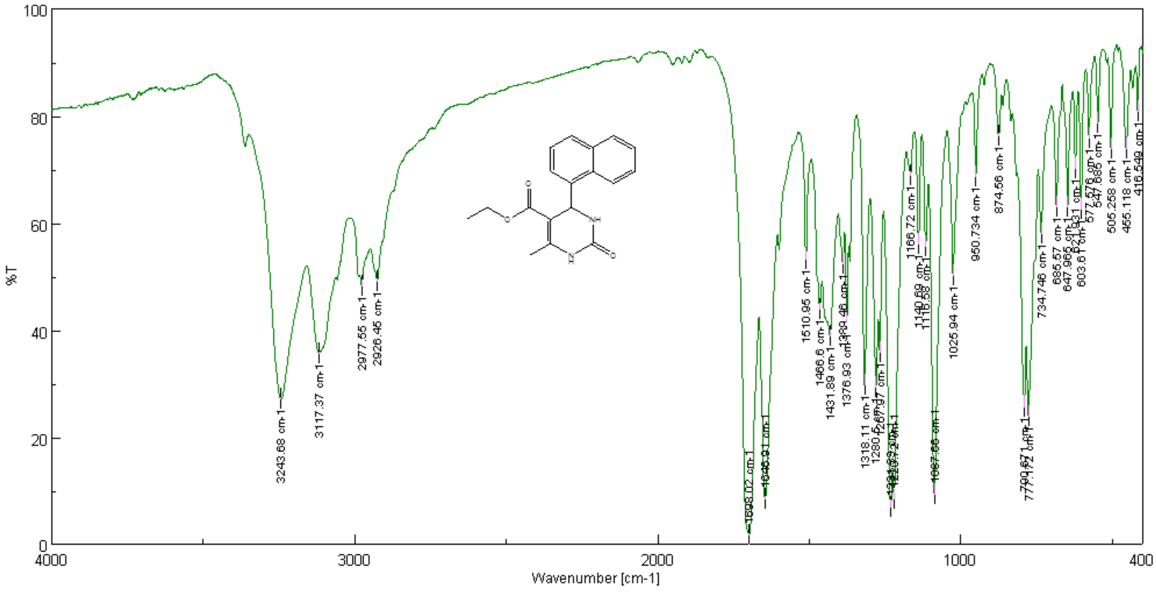

Figure S23. FTIR of 5-(ethoxycarbonyl)-4-(4-naphthalene-1-yl)-6-methyl-3,4-dihydropyrimidin-2(1H)-one.

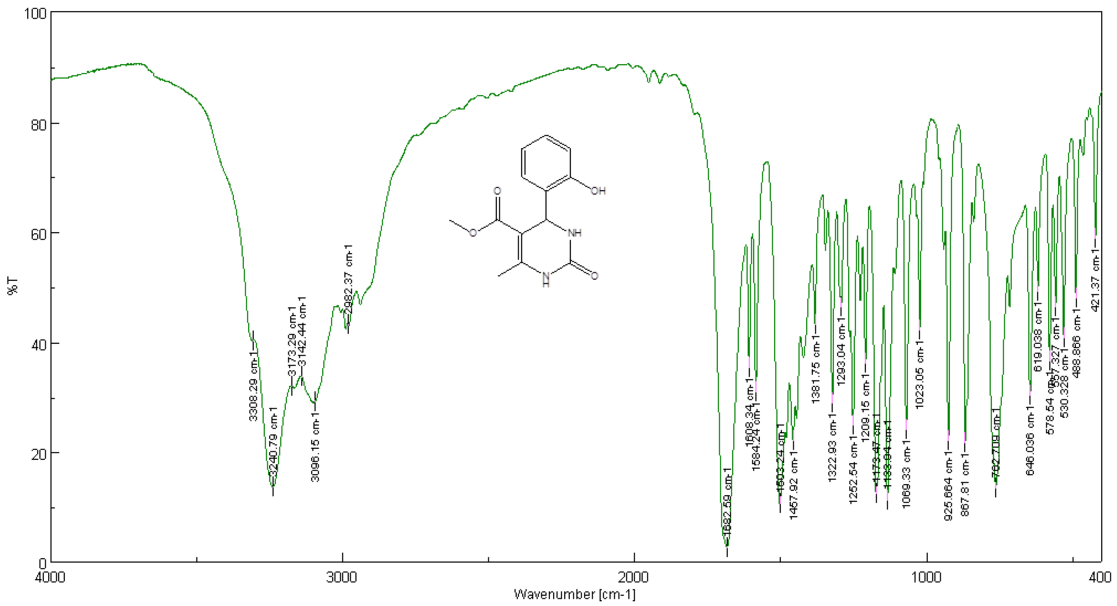

Figure S24. FTIR of 5-(methoxycarbonyl)-6-methyl-4-(2-hydroxyphenyl)-3,4-dihydropyrimidin-2(1H)-one.

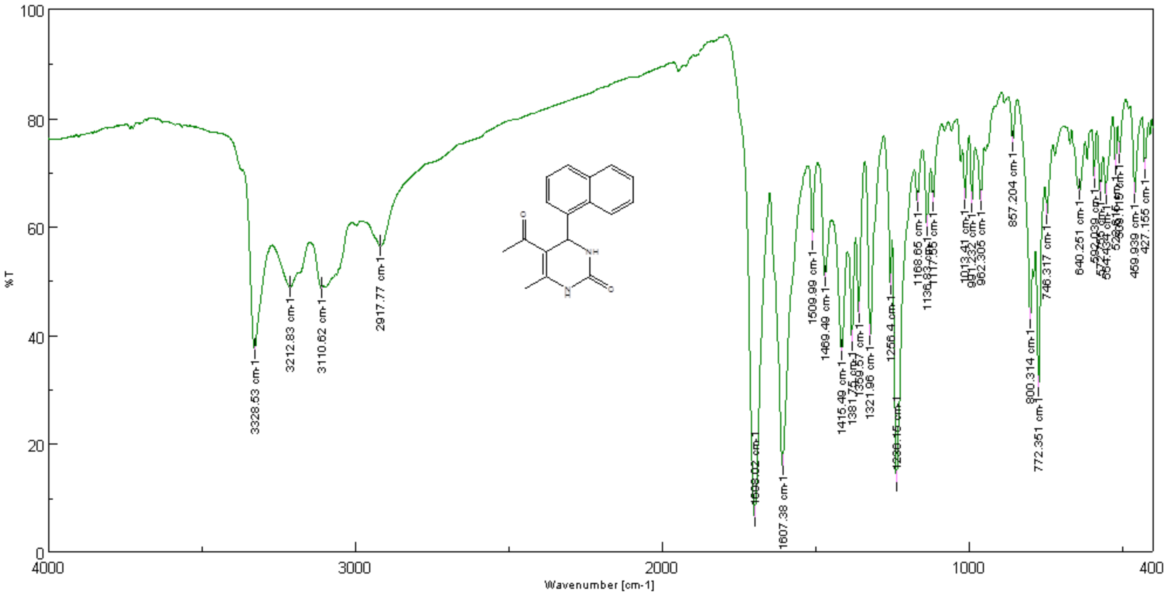

Figure S25. FTIR of 5-acetyl-6-methyl-4-(4-naphthalene-1-yl)-3,4-dihydropyrimidin-2(1H)-one.

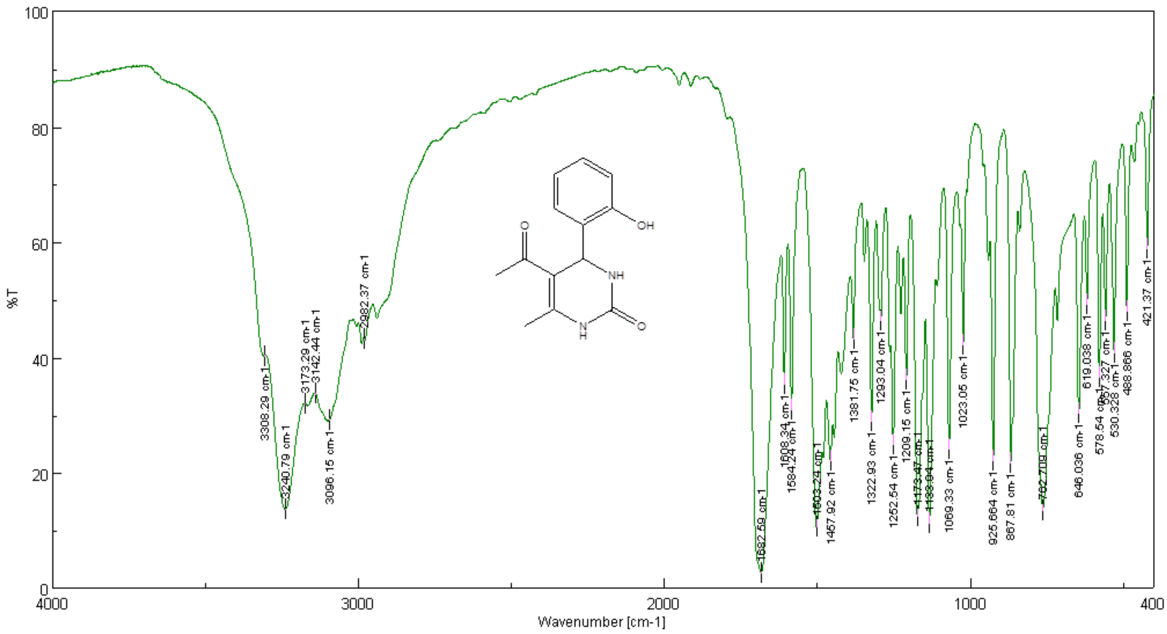

Figure S26. FTIR of 5-acetyl-6-methyl-4-(2-hydroxyphenyl)-3,4-dihydropyrimidin-2(1H)-one.

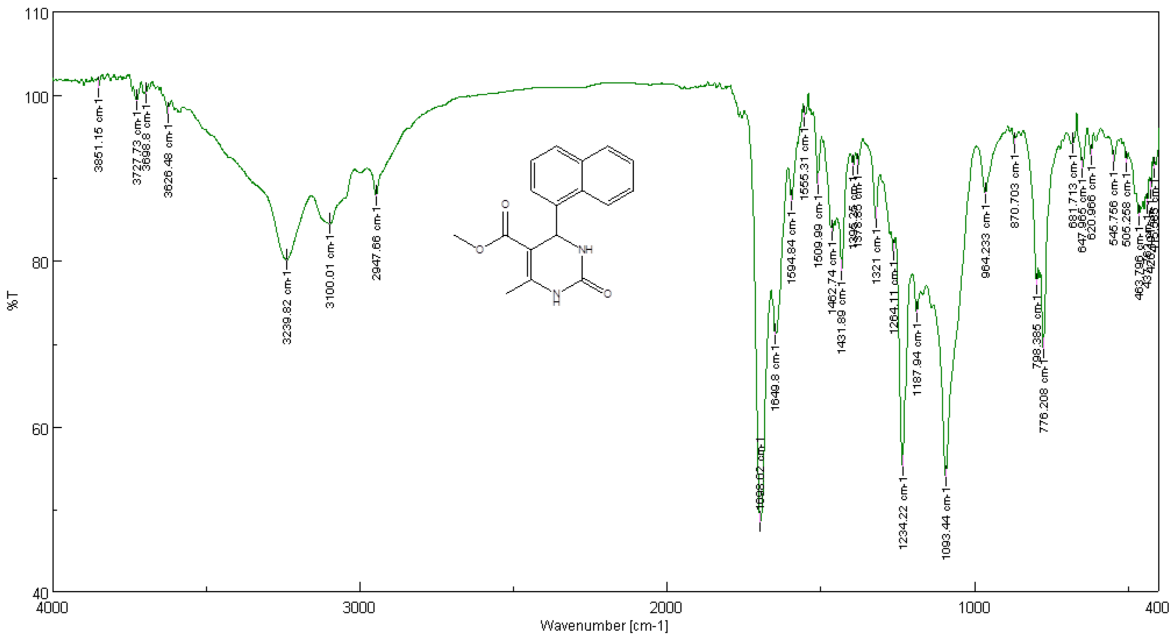

Figure S27. FTIR of 5-methoxycarbonyl-6-methyl-4-(4-naphthalene-1-yl)-3,4-dihydropyrimidin-2(1H)-one.

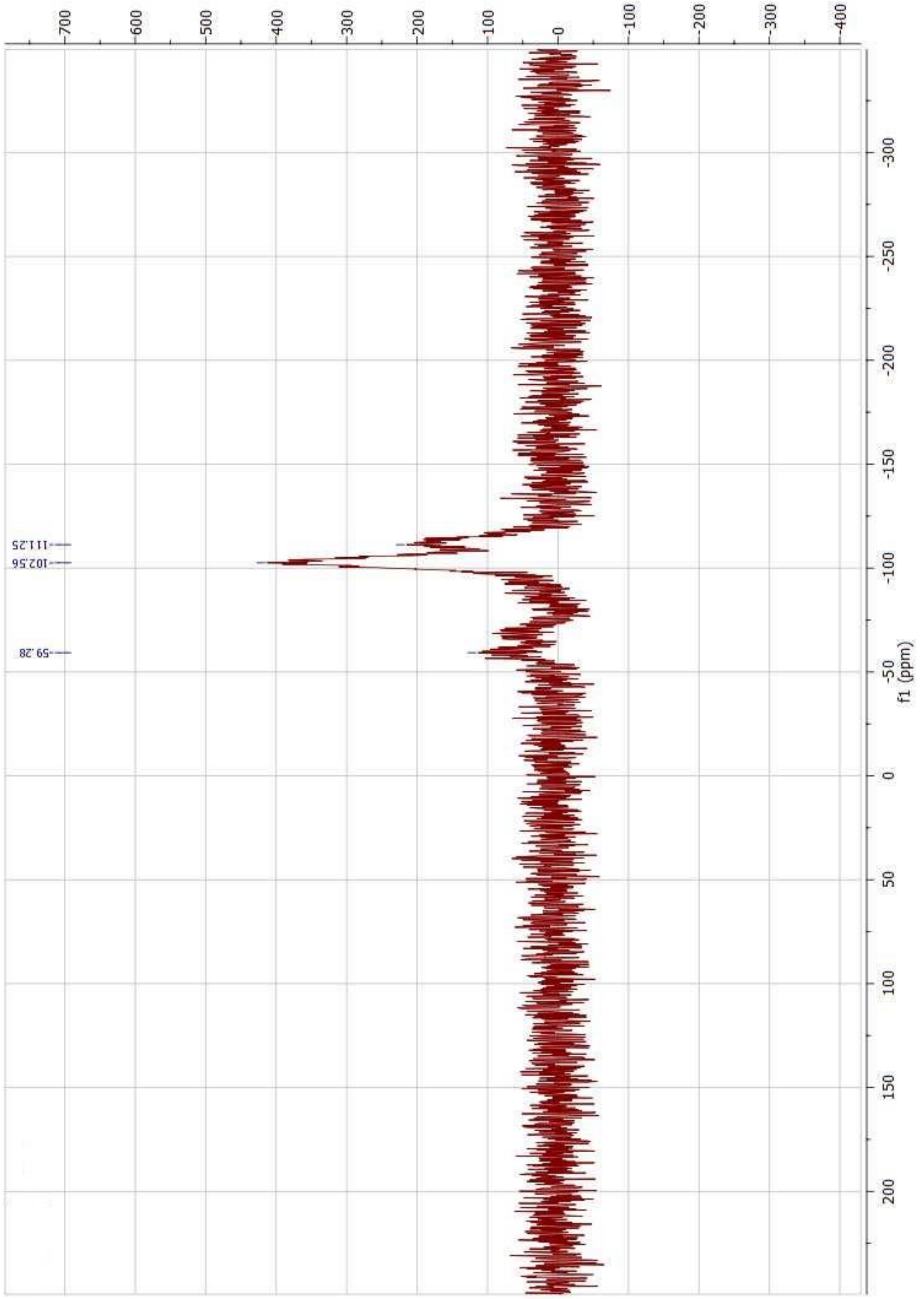

Figure S28. ^{29}Si NMR of Cu@SBA-15 nanostructures.

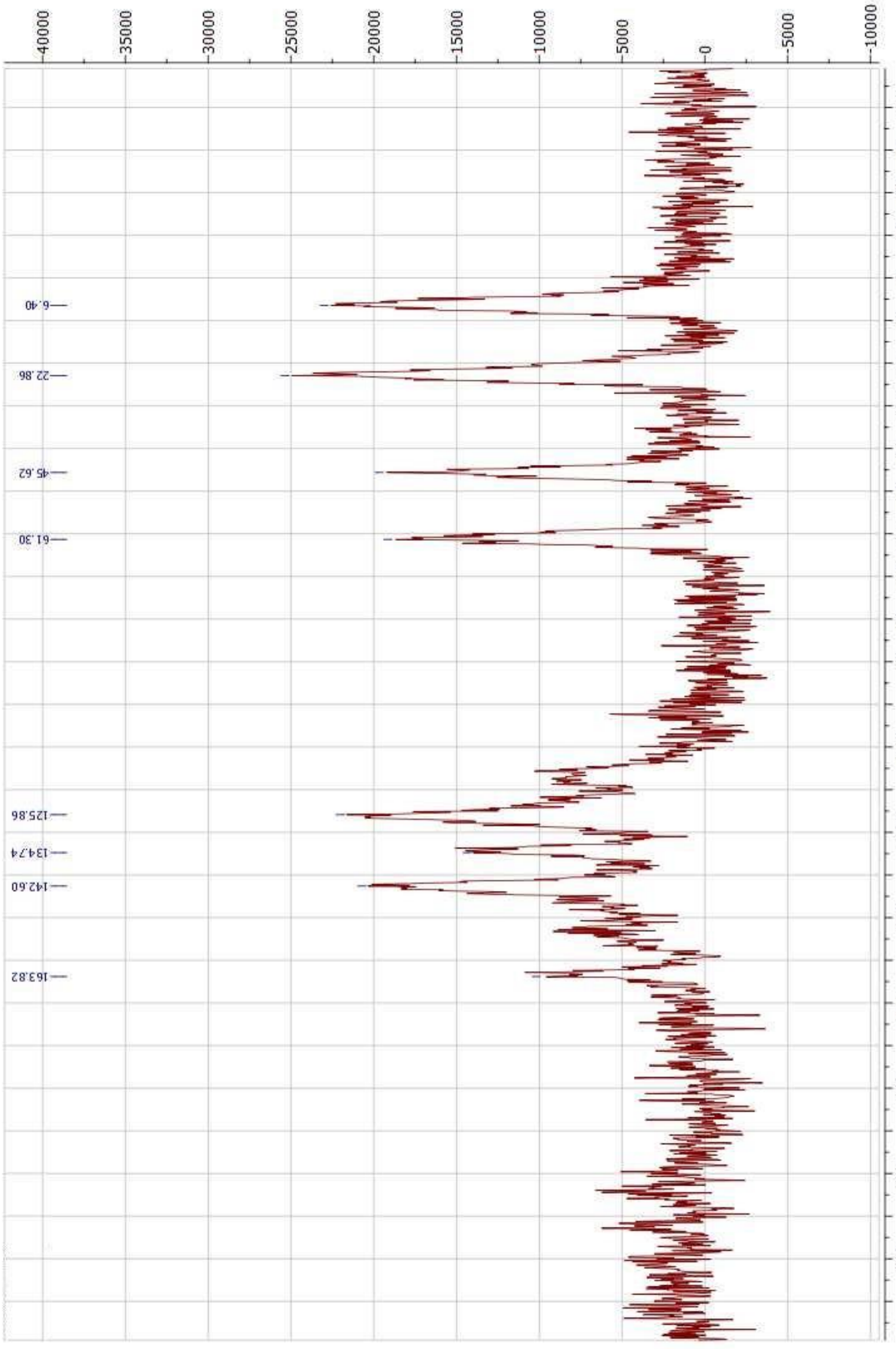

Figure S29. ^{13}C NMR of Cu@SBA-15 nanostructures.
